# A Collective CRISPR-Cas12a Biosensing Platform for Effect-Based Screening of Pesticide Residues

**DOI:** 10.3390/bios16100553

**Published:** 2026-10-02

**Authors:** Xiaoya Bo, Ji Li, Chao Huang, Qun Liu, Mingyue Qi, Qunxian Li, Long Dai, Riran Zhu, Shengyong Ding

**Affiliations:** 1 The First Clinical Medical College, Shandong University of Traditional Chinese Medicine, Jinan 250014, Chinazhuriran@163.com (R.Z.); 2 Affiliated Hospital of Shandong University of Traditional Chinese Medicine, Jinan 250014, China

**Keywords:** pesticide residue screening, effect-based sensing, collective biosensing, CRISPR-Cas12a, click-chemistry

## Abstract

The simultaneous screening of multicomponent pesticide residues, rather than a single specific compound, remains a critical yet challenging frontier in safety surveillance, largely due to the inherent one-to-one recognition bottleneck in conventional aptamer- or antibody-based biosensors. Herein, we develop a collective CRISPR-Cas12a biosensing platform for effect-based screening of pesticide residues by integrating effect-based enzymatic recognition with PAM-independent CRISPR signal amplification. Rather than employing a dedicated aptamer or antibody for each pesticide, the proposed strategy exploits the inhibitory effects of diverse pesticide residues on alkaline phosphatase (ALP) as a common biochemical response. ALP activity inhibition serves as a common trigger, converting the presence of diverse toxicants into the controlled generation of Cu^+^ as a unified biochemical signal via ALP-mediated ascorbic acid production. To transduce this signal into a robust nucleic acid output, we further engineer a CRISPR-Cas12a system that operates completely independent of PAM sequences through click chemistry-mediated covalent ligation of two hairpin fragments. The complete activator strand activated Cas12a trans-cleavage, thereby circumventing the sequence constraints imposed by PAM recognition. The platform exhibits quantitative responses toward TCE, CPF, and Piri, with limits of detection of 22.1 nM, 19.3 nM, and 12.0 nM, respectively. Furthermore, it successfully reports pesticide contamination in a complex real sample matrix, which provides a simple and flexible strategy for preliminary pesticide screening. Importantly, the platform is not intended to convert the collective fluorescence response into the total concentration of diverse pesticide residues; rather, the output reflects the integrated perturbation of the ALP-mediated biochemical pathway under the defined assay conditions. Overall, this work establishes an effect-based collective sensing paradigm that shifts pesticide screening from compound-specific molecular recognition toward integrated biochemical response, providing a flexible strategy for rapid screening and early warning of multicomponent pesticide contamination.

## 1. Introduction

Modern agriculture relies heavily on chemical pesticides to secure global food production, yet the resulting contamination of agricultural products poses escalating threats to ecosystems and human health. This challenge is caused by the fact that real-world contamination rarely involves a single compound; multiple residues often coexist, potentially inducing synergistic toxic effects more severe than those of individual agents [1,2,3]. Consequently, developing analytical tools capable of rapidly indicating the collective biochemical effects associated with multicomponent pesticide contamination would be valuable for preliminary safety screening.

Current instrumental methods, such as HPLC-MS and GC-MS, deliver unparalleled accuracy and sensitivity for reference analysis [4,5,6]. However, their reliance on expensive infrastructure, laborious sample preparation, and skilled operators renders them inadequate for rapid, on-site preliminary screening. Thus, a complementary screening technology capable of rapidly indicating the presence of pesticide-associated biochemical perturbations in samples containing potentially multiple residues would be valuable for preliminary screening before comprehensive instrumental confirmation [4,7]. In response, nucleic acid-based biosensors, particularly those employing functional nucleic acids (FNAs) and isothermal amplification, have emerged as promising alternatives [8]. Leveraging the exquisite programmability of DNA, researchers have constructed highly specific sensing platforms using aptamers, DNAzymes, catalytic hairpin assembly, hybridization chain reaction, and CRISPR systems [9,10,11]. For instance, elegant works have achieved ultrasensitive detection of individual pesticides such as acetamiprid, malathion, and chlorpyrifos.

CRISPR systems, particularly CRISPR-Cas12a, have attracted considerable attention because of their programmable target recognition and robust signal amplification through trans-cleavage activity, although their activation traditionally depends on sequence constraints such as the PAM requirement [12,13,14,15,16,17,18]. Nevertheless, a fundamental limitation of these designs is that they nearly all rely on the “one-aptamer or one-antibody to one-target” recognition paradigm, which not only restricts detection to a single analyte per assay but also imposes stringent sequence constraints that complicate the integration of non-nucleic-acid targets [19,20]. This dual bottleneck makes it both economically and technically prohibitive for a wide range of pesticides. What is urgently needed is a collective sensing strategy that simultaneously overcomes both the specificity-driven recognition and the sequence-dependent amplification barrier, thereby converting the presence of diverse pesticide family into a single, measurable signal for rapid early warning of overall toxicity.

**Scheme 1:**
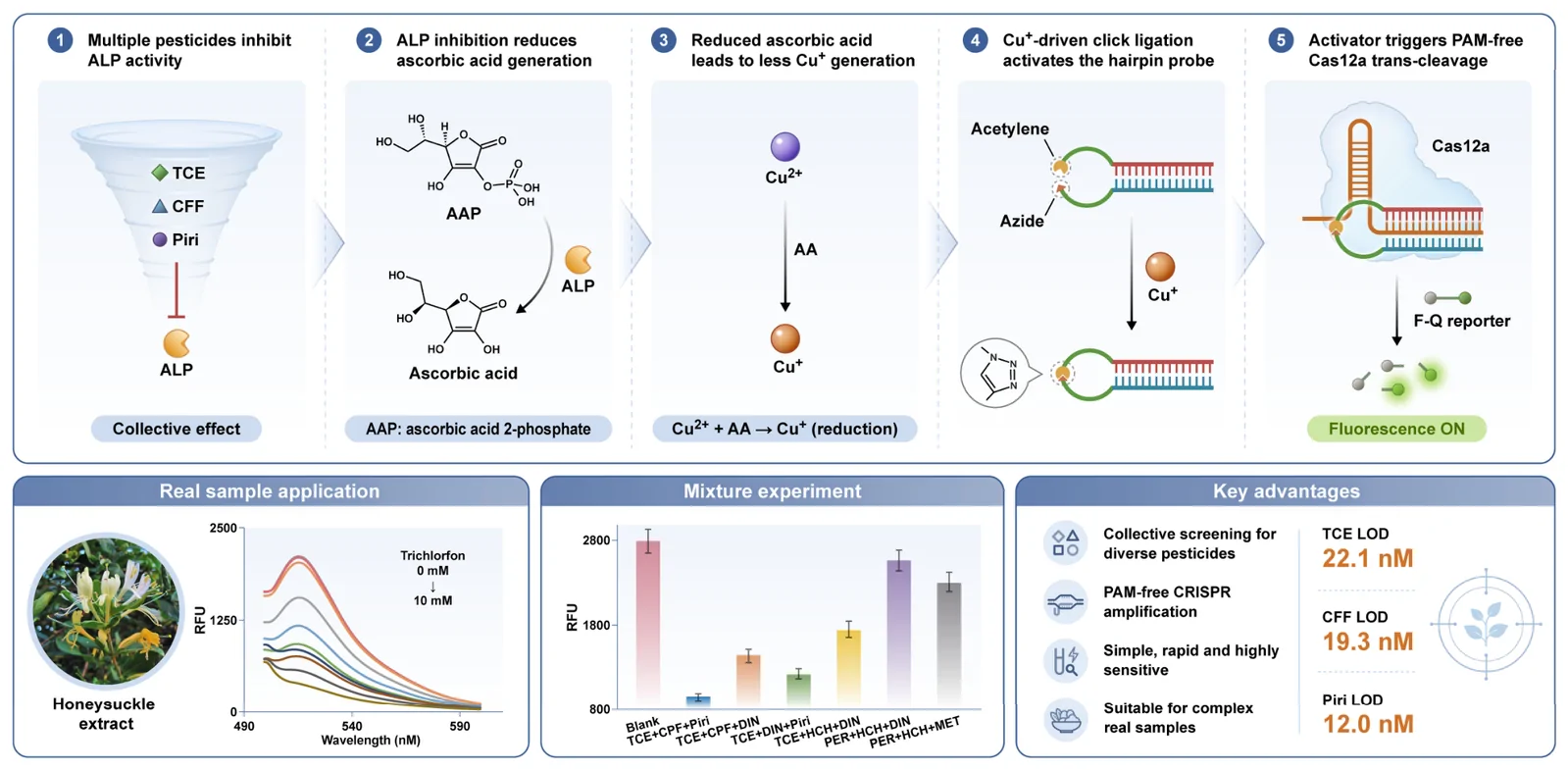
Schematic illustration of the collective CRISPR-Cas12a biosensing platform for effect-based screening of pesticide residues.

In this work, we address this challenge by shifting from molecular recognition to biochemical effect (Scheme 1). We exploit the observation that a wide range of pesticides, including organophosphates, synthetic pyrethroids, and phenoxycarboxylic acid herbicides, share a common inhibitory effect on alkaline phosphatase (ALP) activity [21,22,23]. By employing ALP as a broad-spectrum biological indicator, we transduce overall pesticide toxicity into a differential output of enzymatic activity, specifically, the generation of ascorbic acid and subsequent reduction of Cu^2+^ to Cu^+^ [24,25,26,27]. To couple this chemical event to a powerful nucleic acid amplification readout without being constrained by PAM sequences, we introduce a PAM-free activation mechanism: a Cu-catalyzed click reaction covalently joins two rationally designed hairpin fragments, generating the complete double-stranded activator required for Cas12a trans-cleavage, entirely independent of any PAM motif. This design liberates the CRISPR system from sequence restrictions while seamlessly linking the ALP-inhibition signal to the amplification power of CRISPR-Cas12a. Importantly, the present strategy should be distinguished from conventional multiresidue quantitative analysis. Because different pesticides exhibit different inhibitory efficiencies toward ALP, the fluorescence output represents an integrated biochemical response rather than a universal concentration-equivalent value applicable to all pesticide molecules. Accordingly, the proposed platform is designed primarily for effect-based preliminary screening and early warning of pesticide contamination, whereas compound-specific identification and quantitative determination should be performed using established chromatographic or mass-spectrometric methods.

## 2. Experimental Section

### 2.1. Materials and Reagents

All oligonucleotides used in this study were synthesized by Sangon Biotech Co., Ltd. (Shanghai, China) with HPLC purification and are listed below. The sequences of the crRNA, template strand (TS), non-template strand (NTS), and fluorescence-quenched (FQ) reporter probe are summarized as follows:

crRNA (5′-UAAUUUCUACUAAGUGUAGAUCUCAGGGCGGACGAGGUUUAGGAU-3′)

TS (5′-ATCCTAAACCTCGTCCGCCCTGAGCAA-Azide-3′)

NTS (5′-alkyne-GATCTCAGGGCGGACGAGGTTTAGGAT-3′)

FQ reporter (5′-FAM-TTTTTTTTTTTTTTT-BHQ1-3′)

Recombinant LbCas12a, 5× TBE buffer (89 mM Tris, 89 mM boric acid, 2.0 mM EDTA, pH 8.3), and diethylpyrocarbonate (DEPC)-treated water were purchased from Sangon Biotech Co., Ltd. (Shanghai, China). 10× rCutSmart buffer (50 mM CH_3_COOK, 20 mM Tris-acetate, 10 mM Mg(CH_3_COO)_2_, 100 µg/mL recombinant albumin, pH 7.9) was obtained from New England Biolabs (Beijing, China). Ammonium persulfate (APS) and tetramethylethylenediamine (TEMED) were procured from Beyotime Biotechnology (Shanghai, China). DNA marker and 6× loading buffer were sourced from Takara Bio Co., Ltd. (Beijing, China).

Pesticide standards, including trichlorfon, chlorpyrifos, dichlorodiphenyltrichloroethane (DDT), hexachlorocyclohexane (HCH), permethrin, deltamethrin, metolachlor, dinotefuran, acetamiprid, and thiamethoxam, were obtained from ANPEL-TRACE Co., Ltd. (Shanghai, China) or Macklin Biochemical Co., Ltd. (Shanghai, China). Alkaline phosphatase (ALP) was diluted to 40 mU·μL^−1^ in 1× rCutSmart buffer prior to use. All other chemicals were of analytical grade and used without further purification.

### 2.2. Instrumentation

Fluorescence measurements were recorded on an F-320 fluorescence spectrophotometer (Tianjin Gangdong Technology Co., Ltd., Tianjin, China) equipped with a xenon lamp. The excitation and emission slit widths were both set at 5 nm, and the photomultiplier tube voltage was maintained at 700 V. Emission spectra were collected from 500 to 600 nm upon excitation at 488 nm, and the fluorescence intensity at 514 nm was used for quantitative analysis. Polyacrylamide gel electrophoresis (PAGE) was performed using a JY-SCZ9 electrophoresis cell (Beijing Changliu Scientific Instrument Co., Ltd. Beijing, China) coupled with an HX-105 constant-temperature circulating water system and a DYY-6C electrophoresis power supply. Gel imaging was carried out with a Tanon 3500-BR ultraviolet imaging system (Shanghai Tianneng Life Science Co., Ltd. Shanghai, China).

### 2.3. Preparation of DNA Probes

The TS and NTS single-stranded oligonucleotides were mixed at a molar ratio of 1:1.5 in 1× rCutSmart buffer to a final volume of 10 μL. The mixture was heated at 95 °C for 5 min and then gradually cooled to room temperature to allow annealing and formation of the TS-NTS duplex probe. The annealed probe was stored at 4 °C for immediate use. Other DNA oligonucleotides were subjected to similar annealing procedures when required, and those not used immediately were stored at −20 °C.

### 2.4. Cu^+^-Catalyzed Click Chemistry Reaction

The click chemistry reaction was carried out in a 10 μL system containing TS-NTS probe (0.5 μM, 2 μL), Cu^2+^ (1 mM, 5 μL), ascorbic acid phosphate (AAP, 6 mM, 1 μL), and ALP (40 mU·μL^−1^, 1 μL) in 1× rCutSmart buffer. The mixture was incubated at 37 °C for 2 h to allow ALP-mediated reduction of Cu^2+^ to Cu^+^ and subsequent Cu^+^-catalyzed covalent ligation of the azide- and alkyne-modified DNA fragments.

### 2.5. Formation of CRISPR-Cas12a Ribonucleoprotein (RNP) Complex

Cas12a (10 μM, 0.5 μL) and crRNA (20 μM, 0.5 μL) were mixed with 2 μL of 10× reaction buffer (composed of 10× rCutSmart buffer and 0.1 M DTT at a volume ratio of 8:2) in DEPC-treated water to a final volume of 20 μL. The mixture was incubated at 37 °C for 30 min to allow pre-assembly of the Cas12a-crRNA ribonucleoprotein (RNP) complex.

### 2.6. CRISPR-Cas12a Trans-Cleavage Assay and Fluorescence Detection

After the click chemistry reaction, 2 μL of the pre-formed RNP complex and 5 μL of 10× reaction buffer were added to the 10 μL click reaction mixture, followed by the FQ reporter probe (1 μM, 5 μL). The final volume was adjusted to 50 μL with DEPC-treated water. The resulting mixture was incubated at 37 °C for 30 min to trigger Cas12a-mediated trans-cleavage of the FQ reporter, generating a fluorescence signal. Following incubation, fluorescence spectra were immediately recorded using the fluorescence spectrophotometer. The emission intensity at 514 nm was extracted for quantitative analysis.

### 2.7. Non-Denaturing Polyacrylamide Gel Electrophoresis (PAGE)

To verify the click ligation and CRISPR activation, 5 μL of the reaction mixture was mixed with 1 μL of 6× loading buffer, and 5 μL of the mixture was loaded onto a 12% non-denaturing polyacrylamide gel. The gel was prepared by mixing 5 mL of 5× TBE buffer, 7.5 mL of 40% acrylamide/bisacrylamide solution (19:1), 12.5 mL of deionized water, 18 μL of TEMED, and 180 μL of APS (0.1 g·mL^−1^). Electrophoresis was performed in 0.5× TBE running buffer at a constant current of 30 mA and a constant temperature of 15 °C for 50 min. After electrophoresis, the gel was stained with SYBR Gold dye for 30 min in the dark at room temperature and imaged using the Tanon 3500-BR UV imaging system.

### 2.8. Pesticide Inhibition Assay

For pesticide detection, different concentrations of pesticide standards were first mixed with ALP, Cu^2+^, and the TS-NTS probe in 1× rCutSmart buffer. The mixture was pre-incubated at 37 °C for 1 h to allow pesticide-induced inhibition of ALP activity. Subsequently, AAP was added to initiate ALP-mediated dephosphorylation and the subsequent reduction of Cu^2+^ to Cu^+^. After incubation, the resulting reaction mixture was directly subjected to the Cu^+^-mediated click ligation reaction and subsequent CRISPR-Cas12a trans-cleavage assay as described above. The fluorescence intensity at 514 nm was recorded after the CRISPR reaction. Because pesticide-induced inhibition of ALP decreases the production of ascorbic acid and consequently reduces Cu^+^ generation and activator formation, the fluorescence response decreased with increasing pesticide concentration.

For the three representative pesticides, trichlorfon (TCE), chlorpyrifos (CPF), and pirimiphos-methyl (Piri), concentration-response curves were constructed by plotting fluorescence intensity against the corresponding pesticide concentration. These calibration relationships were used only for the individual pesticides investigated in this study and were not intended to represent a universal calibration relationship applicable to chemically diverse pesticide mixtures.

### 2.9. Analysis of Pesticide Mixtures

To evaluate the collective sensing capability of the proposed platform toward multicomponent pesticide contamination, different pesticide mixtures were prepared by combining representative pesticides at the tested concentrations. The mixtures included TCE + CPF + Piri, TCE + CPF + DIN, TCE + DIN + Piri, TCE + HCH + DIN, PER + HCH + DIN, and PER + HCH + MET. Each mixture was incubated with ALP, Cu^2+^, and the TS-NTS probe under the same conditions used for individual pesticide detection. Following AAP addition and incubation, the reaction mixtures were subjected to the click-CRISPR cascade, and the fluorescence intensity was measured at 514 nm. A pesticide-free reaction was used as the blank control.

The fluorescence responses of the pesticide mixtures were compared with that of the blank control to evaluate whether the proposed effect-based sensing platform could generate an integrated response to multicomponent pesticide contamination. The mixture responses were interpreted as collective biochemical responses rather than as quantitative estimates of the concentrations of individual pesticide components.

### 2.10. Real Sample Analysis in Traditional Chinese Medicine Matrix

To preliminarily evaluate the performance of the proposed strategy in a complex real-world matrix, Lonicerae Japonicae Flos (honeysuckle) was selected as a representative Traditional Chinese Medicine (TCM) matrix. Briefly, 1 g of powdered honeysuckle sample was mixed with 10 mL of methanol and subjected to ultrasonic extraction for 30 min. The mixture was then centrifuged at 5000 rpm for 10 min, and the supernatant was collected. The obtained extract was spiked with different concentrations of trichlorfon as a model pesticide, and the spiked samples were analyzed following the pesticide inhibition assay procedure described above. The recovery experiments were performed to preliminarily evaluate matrix compatibility, recovery, and precision under the tested conditions.

## 3. Results and Discussion

### 3.1. Design and Validation of the PAM-Free CRISPR-Cas12a Activation Strategy

**Figure 1:**
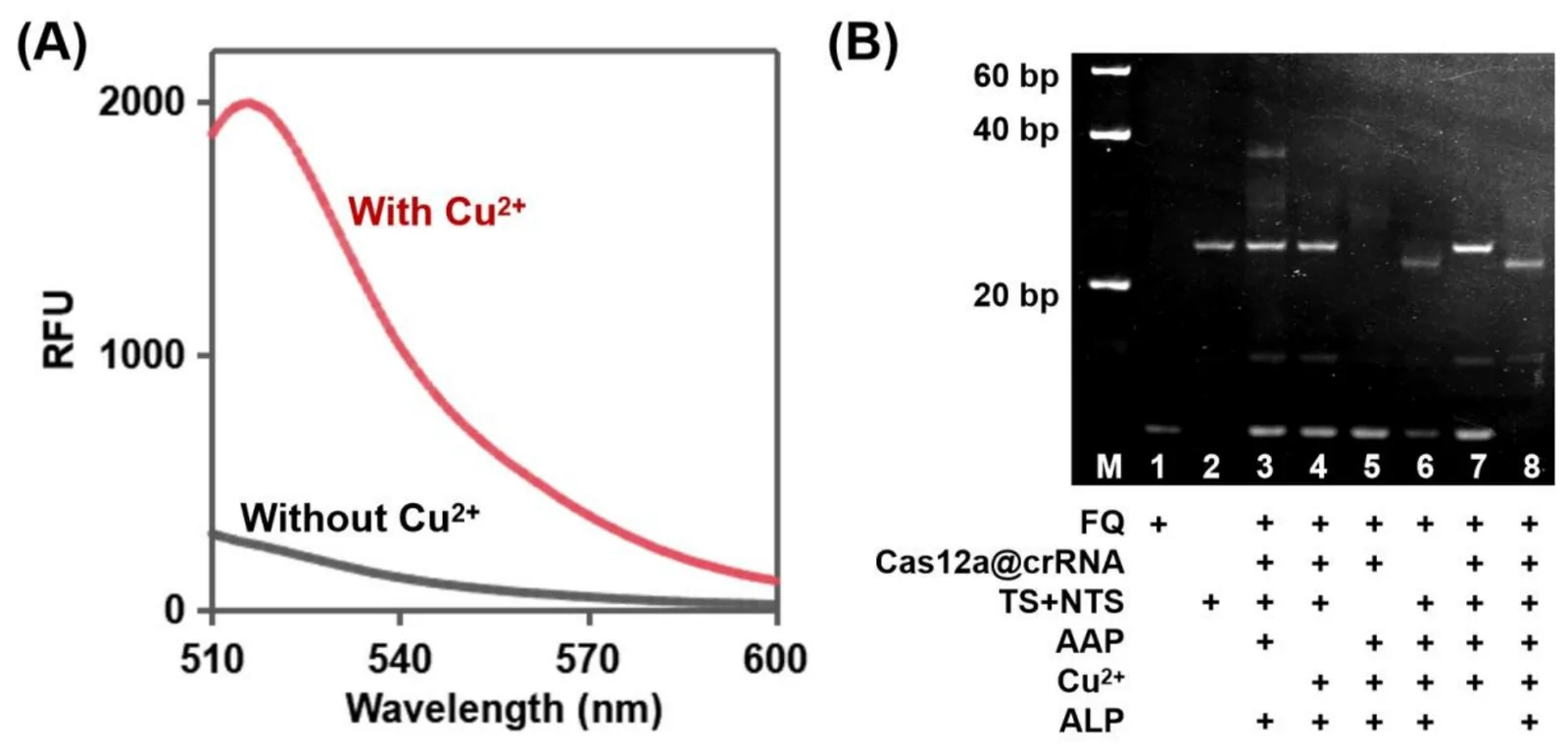
Feasibility verification of this collective CRISPR-Cas12a biosensing platform. (**A**) Fluorescence spectra of the system in the presence of Cu^2+^ and without Cu^2+^. (**B**) PAGE image: (1) FQ, (2) TS + NTS, (3) Control without Cu^2+^, (4) Control without AAP, (5) Control without TS + NTS, (6) Control without Cas12a@crRNA, (7) Control without ALP, (8) Positive control.

The sensing mechanism was designed to integrate effect-based enzymatic recognition with Cu^+^-mediated DNA ligation and PAM-free CRISPR-Cas12a signal amplification. As illustrated in Figure 1, pesticide-induced inhibition of ALP decreases the enzymatic conversion of AAP to ascorbic acid, thereby suppressing the reduction of Cu^2+^ to Cu^+^. Because Cu^+^ is required for the click chemistry-mediated ligation of the azide- and alkyne-modified DNA fragments, reduced Cu^+^ generation is expected to reduce the formation of the complete Cas12a activator and a lower fluorescence output.

We first evaluated the feasibility of this signal-conversion cascade. As shown in Figure 1A, the complete reaction system produced a markedly stronger fluorescence response in the presence of Cu^2+^ than in its absence, indicating that Cu^2+^-dependent chemical conversion was essential for downstream signal generation. The control experiments further confirmed the requirement of each component in the cascade. Removal of AAP, TS-NTS, ALP, or the Cas12a-crRNA complex suppressed the fluorescence, demonstrating that ALP-mediated substrate conversion, Cu^+^-dependent DNA ligation, and Cas12a trans-cleavage are all indispensable for signal generation.

The high fluorescence observed in the pesticide-free blank is an intrinsic feature of the turn-off sensing architecture rather than nonspecific background. In the absence of pesticide, ALP remains active and efficiently converts AAP to ascorbic acid, promoting Cu^+^ generation, DNA click ligation, Cas12a activator formation, and subsequent cleavage of the fluorescent reporter. The blank therefore represents the maximally activated state of the sensing cascade.

The PAGE results provided further evidence for the successful assembly of the DNA activator (Figure 1B). Compared with the individual TS-NTS components, the complete reaction generated a slower-migrating DNA species, consistent with the formation of the covalently ligated product. Importantly, the formation of this product was accompanied by an enhanced fluorescence response, supporting the functional coupling between click ligation and Cas12a activation.

Taken together, these results establish a sequential signal-conversion pathway in which enzymatic activity is first translated into Cu^+^ generation, followed by covalent DNA assembly and PAM-independent Cas12a activation. This design effectively separates the upstream biochemical recognition event from the sequence-dependent requirements traditionally associated with Cas12a activation.

For subsequent quantitative comparison, the fluorescence intensity at 514 nm, corresponding to the selected emission wavelength of the FAM reporter under the experimental conditions, was used throughout the study.

### 3.2. Optimization of the Click-CRISPR Signal Conversion System

**Figure 2:**
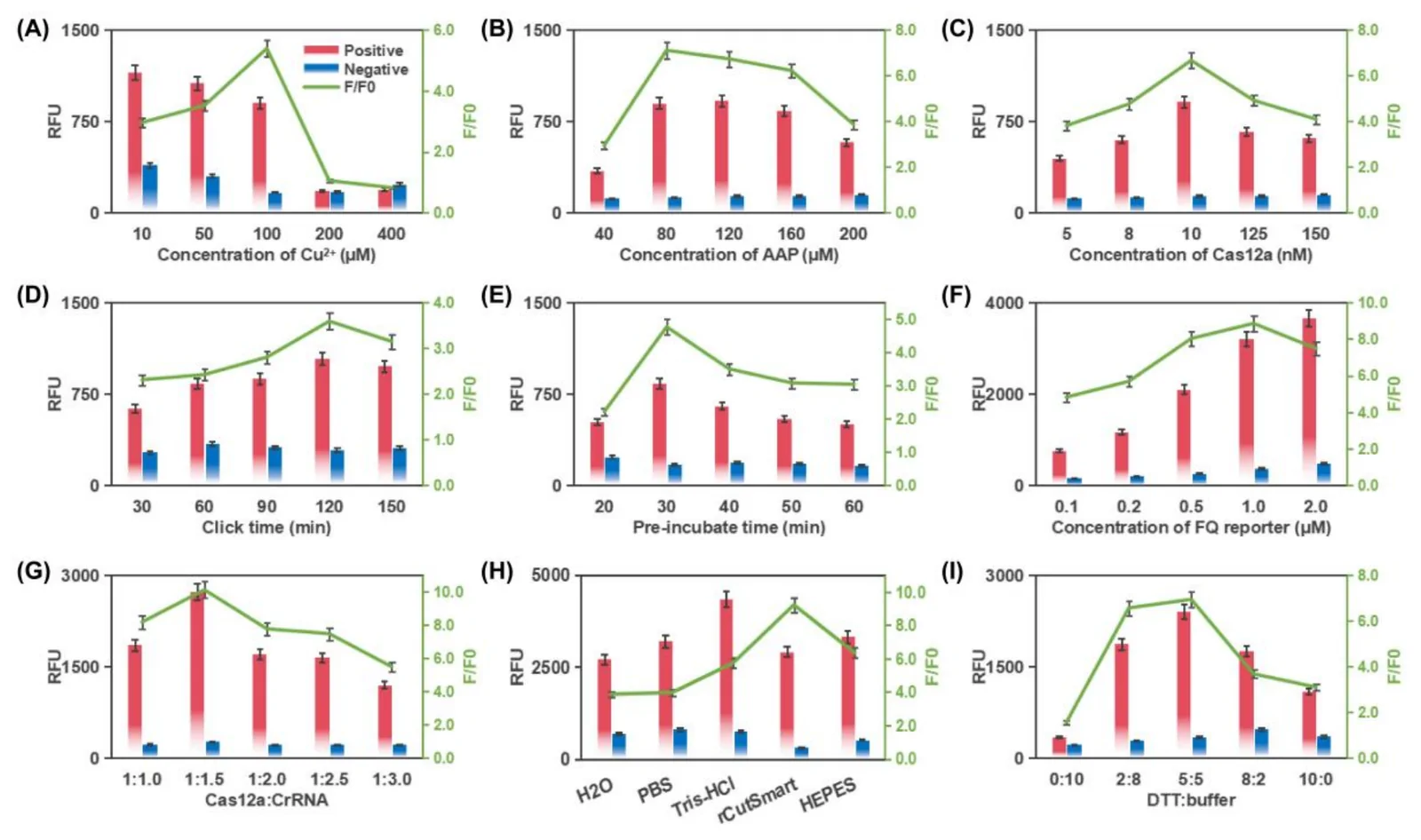
Optimization of key parameters for this collective CRISPR-Cas12a biosensing platform. (**A**) The concentration of Cu^2+^, (**B**) The concentration of AAP, (**C**) The concentration of Cas12a, (**D**) Click time, (**E**) Pre-incubate time, (**F**) The concentration of FQ reporter, (**G**) Cas12a:CrRNA, (**H**) Buffers, (**I**) DTT:buffer. the histogram illustrated the fluorescence intensity variation in the system across different conditions. The orange line chart illustrated the trend of F/F0 value. Error bars represented the standard deviation derived from three independent experiments.

After validating the feasibility of the proposed cascade, the major reaction parameters were systematically optimized to maximize the fluorescence response and signal-to-background ratio (Figure 2).

First, the concentrations of Cu^2+^ and AAP were optimized because Cu^+^ generated through the ALP-mediated reaction provides the chemical basis for the subsequent click ligation, the concentrations of Cu^2+^ and AAP were optimized to obtain an appropriate balance between signal generation and background suppression. As shown in Figure 2A, the F/F_0_ ratio increased from 2.96 to 5.39 as the Cu^2+^ concentration increased from 10 to 100 μM, whereas a pronounced decrease was observed at higher concentrations, with F/F_0_ values of approximately 1.04 and 0.82 at 200 and 400 μM, respectively. Therefore, 100 μM Cu^2+^ was selected for subsequent experiments. A similar trend was observed for AAP (Figure 2B). The F/F_0_ ratio increased from approximately 2.97 to 7.11 when the AAP concentration increased from 40 to 80 μM, followed by a decrease at higher concentrations. Accordingly, 80 μM AAP was selected as the optimal concentration. These results indicate that efficient signal conversion requires a balanced Cu^2+^/reducing-substrate ratio rather than simply maximizing Cu^+^ generation.

The CRISPR-Cas12a parameters were subsequently optimized. The fluorescence response increased from an F/F_0_ value of approximately 1.31 at 5 nM Cas12a to 2.49 at 10 nM, followed by a decline at higher Cas12a concentrations (Figure 2C). Thus, 10 nM Cas12a was selected. The Cas12a:crRNA ratio was further optimized, and the highest response was obtained at a ratio of 1:1.5, with an F/F_0_ value of approximately 10.12 (Figure 2G). These results suggest that excessive Cas12a or an imbalanced RNP composition may compromise the efficiency of target-induced trans-cleavage.

Reaction kinetics were then investigated. The click-ligation signal gradually increased with incubation time and reached a maximum at 120 min, corresponding to an F/F_0_ value of approximately 3.59, whereas prolonged incubation to 150 min resulted in a decrease (Figure 2D). For the subsequent CRISPR reaction, the fluorescence response reached its maximum after 30 min and decreased upon further extension of the incubation time (Figure 2E). These results indicate that prolonged incubation does not necessarily improve signal output and may instead increase background reactions or compromise the stability of the reaction components.

The reporter concentration, reaction buffer, and DTT composition were finally optimized. The strongest fluorescence response was obtained with 1 μM FQ reporter (Figure 2F). Among the tested buffers, rCutSmart generated the highest F/F_0_ value of approximately 9.22 (Figure 2H), confirming the importance of an appropriate ionic environment for Cas12a trans-cleavage. The optimal DTT:buffer ratio was 2:8, which generated an F/F_0_ value of approximately 6.57 (Figure 2I). On the basis of these results, the optimized reaction conditions were used for subsequent analytical studies.

### 3.3. Analytical Performance Toward Representative Organophosphorus Pesticides

We next evaluated the analytical performance of the proposed collective CRISPR-Cas12a platform using three representative organophosphorus pesticides with different chemical structures, namely TCE, CPF, and Piri. These pesticides were selected to determine whether the effect-based sensing mechanism could provide quantitative responses toward structurally diverse compounds without introducing individual recognition elements.

**Figure 3:**
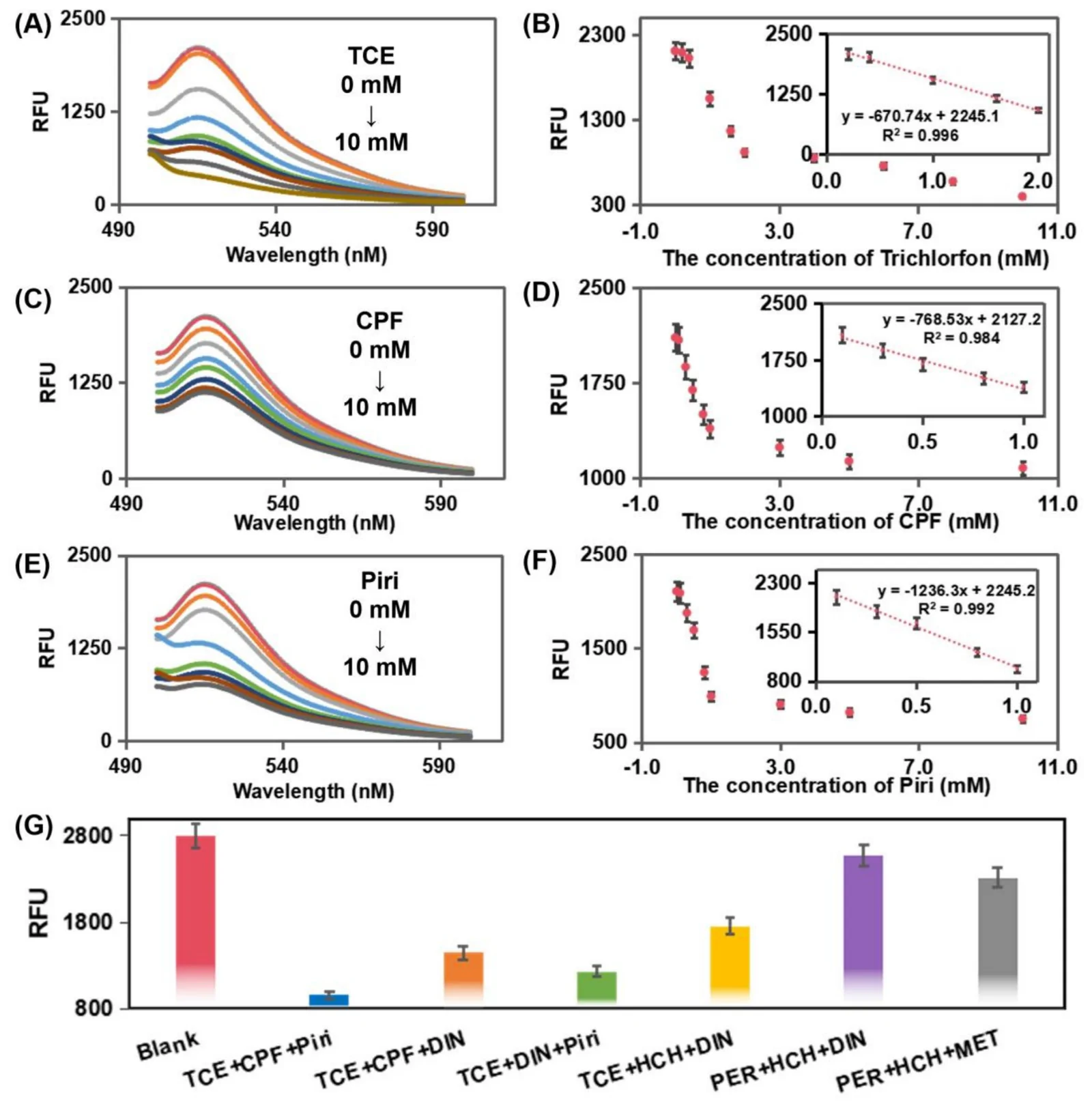
(**A**) Fluorescence spectra of TCE at different concentrations. (**B**) Linear relationships between TCE concentration and fluorescence intensity. (**C**) Fluorescence spectra of CPF at different concentrations. (**D**) Linear relationships between CPF concentration and fluorescence intensity. (**E**) Fluorescence spectra of Piri at different concentrations. (**F**) Linear relationships between Piri concentration and fluorescence intensity. (**G**) The Collective response of the platform toward structurally diverse pesticides. Error bars represent the standard deviation of three parallel measurements. The colored curves represent fluorescence spectra of three representative organophosphorus pesticides at different concentrations, ranging from 0 to 10 mM from top to bottom.

As shown in Figure 3A, increasing TCE concentrations resulted in a progressive decrease in fluorescence intensity. The fluorescence intensity decreased continuously as the TCE concentration increased, reflecting the inhibition of ALP and the consequent suppression of Cu^+^-dependent DNA ligation and Cas12a activation. A good linear relationship was obtained within the investigated concentration range, with a regression coefficient of R^2^ = 0.996 (Figure 3B). The corresponding limit of detection (LOD) was calculated to be 22.1 nM.

A similar concentration-dependent response was observed for CPF (Figure 3C). Increasing CPF concentrations progressively reduced the fluorescence intensity, and the corresponding calibration curve showed a good linear correlation, with R^2^ = 0.984 (Figure 3D). The LOD for CPF was determined to be 19.3 nM.

The platform also responded quantitatively to Piri (Figure 3E). Compared with TCE and CPF, Piri produced a steeper concentration-dependent decrease in fluorescence, as reflected by the slope of the calibration curve. A strong linear relationship was obtained with R^2^ = 0.992 (Figure 3F), and the LOD was calculated to be 12.0 nM.

The different response slopes observed for the three pesticides are consistent with the effect-based nature of the sensing mechanism. Specifically, the platform does not directly recognize pesticide molecular structures; instead, the analytical signal depends on the ability of each pesticide to inhibit ALP. Therefore, pesticides with different inhibitory potencies toward ALP are expected to exhibit different response factors even when measured under identical conditions.

Importantly, the quantitative responses obtained for TCE, CPF, and Piri demonstrate that the sensing strategy is not restricted to a single pesticide-recognition element. Instead, different pesticide molecules can be converted into a common biochemical signal through ALP inhibition and subsequently amplified by the click-CRISPR cascade. This feature provides the fundamental basis for collective pesticide residue screening.

The differential fluorescence responses observed for the three pesticides are consistent with their different effects on the common ALP-mediated biochemical pathway. However, the present study did not directly determine the residual ALP activity for each pesticide concentration and therefore does not establish a universal quantitative conversion between ALP inhibition and downstream fluorescence. Instead, the results demonstrate the functional coupling between pesticide-induced enzymatic perturbation and the downstream click-CRISPR signal output under the standardized assay conditions.

### 3.4. Collective Response Toward Structurally Diverse Pesticides

To further investigate the collective sensing capability of the proposed platform, a series of pesticides with different chemical structures were evaluated under identical experimental conditions. As shown in Figure 3G, the fluorescence responses varied substantially among the tested pesticides. TCE produced the strongest signal suppression, resulting in a fluorescence intensity of approximately 954.9 a.u., whereas CPF generated a moderate response with a fluorescence intensity of approximately 1444.6 a.u. In contrast, the neonicotinoid pesticides dinotefuran, acetamiprid, and thiamethoxam produced relatively weak responses, with fluorescence intensities of approximately 2492.3, 2484.4, and 2377.0 a.u., respectively. HCH, DDT, permethrin, deltamethrin, and metolachlor also exhibited different degrees of signal suppression.

This differential response pattern is an inherent characteristic of the effect-based sensing mechanism. Because the platform relies on pesticide-induced perturbation of ALP activity rather than compound-specific molecular recognition, the fluorescence output reflects the relative inhibitory effect of each pesticide on the common enzymatic pathway. Therefore, the same fluorescence intensity does not necessarily correspond to the same concentration across different pesticide compounds. The platform should consequently not be regarded as a universal quantitative assay with an identical response factor for all pesticide molecules. Instead, it functions as a collective biochemical-response sensor that translates chemically diverse perturbations into a common fluorescence output.

To further determine whether the platform could respond to multicomponent contamination, several pesticide mixtures were subsequently examined (Figure 3G). A pronounced fluorescence decrease was observed for the tested mixtures compared with the blank control, although the magnitude of signal suppression varied substantially among different combinations.

The mixture of TCE + CPF + Piri generated the strongest signal suppression, with a fluorescence response of approximately 900 a.u., markedly lower than the blank control (~2800 a.u.). Other combinations, including TCE + CPF + DIN, TCE + DIN + Piri, and TCE + HCH + DIN, also produced substantially reduced fluorescence responses, with signals of approximately 1500, 1250, and 1750 a.u., respectively. In contrast, PER + HCH + DIN and PER + HCH + MET generated weaker overall responses, with fluorescence intensities of approximately 2550 and 2300 a.u., respectively.

Because the proposed platform relies on effect-based perturbation of ALP activity followed by a Cu^+^-dependent DNA assembly and CRISPR-Cas12a signal amplification cascade, substances capable of affecting any of these biochemical processes may theoretically influence the final fluorescence response. For example, metal ions, chelating agents, redox-active compounds, or other enzyme-modulating substances could potentially interfere with the assay. EDTA and Pb^2+^, as suggested by the reviewer, represent mechanistically relevant examples because they may perturb metal-ion-dependent biochemical processes. However, their potential effects should be considered in the context of the intended application and sample matrix, since these substances are not themselves representative pesticide residues. Moreover, the possible interferents encountered in complex food, herbal, environmental, or biological matrices are diverse, and a finite interference panel cannot completely exclude all potential matrix effects.

These results provide direct experimental evidence that the proposed platform can translate multicomponent pesticide contamination into a unified fluorescence output. Importantly, the magnitude of the response varied according to the composition of the mixture, which is consistent with the different inhibitory activities of individual pesticides toward ALP.

The mixture experiments also highlight an important distinction between collective response and compound-specific quantification. Because the assay measures the integrated perturbation of the ALP-Cu^+^-click-CRISPR cascade, the fluorescence signal generated by a mixture cannot be directly converted into the absolute concentration of each individual pesticide without appropriate calibration. Nevertheless, the ability to distinguish pesticide-containing mixtures from the blank demonstrates the potential of this strategy for rapid preliminary screening of complex contamination.

Notably, the present experiments demonstrate collective effects but do not by themselves establish synergistic or antagonistic interactions among the pesticides. Such conclusions would require dedicated mixture-toxicity models and systematic concentration-response experiments. Therefore, the current platform is more appropriately described as an effect-based collective screening method rather than a method for mechanistic assessment of pesticide mixture interactions.

### 3.5. Preliminary Validation in a Traditional Chinese Medicine Matrix

To evaluate the practical applicability of the proposed biosensing platform, honeysuckle (Lonicerae Japonicae Flos) was selected as a representative Traditional Chinese Medicine matrix. Honeysuckle was extracted with methanol by ultrasonic treatment, followed by centrifugation, and the resulting supernatant was spiked with different concentrations of TCE. The spiked samples were subsequently analyzed using the optimized ALP-Cu^+^-click-CRISPR cascade.

As shown in Table 1, satisfactory recoveries were obtained at all three tested concentrations. The three spike levels were selected to represent low, intermediate, and relatively high concentrations within the experimentally validated response range, providing a preliminary assessment of recovery and precision in the honeysuckle matrix. At a spiked concentration of 0.2 mM, the recoveries were 96.45%, 103.02%, and 107.06%, corresponding to a mean recovery of 102.18% and an RSD of 5.24%. At 1.0 mM, the recoveries were 103.43%, 98.25%, and 107.57%, with a mean recovery of 103.08% and an RSD of 4.53%. At 2.0 mM, the recoveries were 92.33%, 94.29%, and 98.40%, corresponding to a mean recovery of 95.01% and an RSD of 3.26%.

The recoveries ranging from 95.01% to 103.08%, together with RSD values below 5.3%, indicate satisfactory accuracy and precision in the honeysuckle matrix. These results suggest that the methanolic plant extract did not cause substantial loss of analytical recovery under the tested conditions with the sequential ALP-mediated biochemical conversion, Cu^+^-dependent click ligation, or downstream Cas12a trans-cleavage.

More importantly, the successful detection of spiked TCE in a complex plant-derived matrix demonstrates that the effect-based sensing cascade can tolerate matrix complexity and retain its analytical performance outside a simple buffer system. This finding supports the potential use of the proposed method as a rapid preliminary screening tool for pesticide contamination in Traditional Chinese Medicine materials, while confirmatory chromatographic analysis would remain necessary for compound-specific identification and regulatory quantification.

## 4. Conclusions

In summary, we developed a collective CRISPR-Cas12a biosensing platform for effect-based screening of pesticide residues by integrating ALP-mediated biochemical recognition, Cu^+^-catalyzed click chemistry, and PAM-independent CRISPR-Cas12a signal amplification. Rather than relying on a conventional one-recognition-element-to-one-analyte strategy, the proposed platform converts pesticide-induced inhibition of ALP into a common chemical signal, which is subsequently transformed into a nucleic-acid-based fluorescence output through click-mediated DNA assembly and Cas12a trans-cleavage.

The platform exhibited quantitative responses toward three representative organophosphorus pesticides, TCE, CPF, and Piri, with LODs of 22.1, 19.3, and 12.0 nM, respectively. The different response magnitudes observed for structurally diverse pesticides further demonstrated that the sensing output reflects their differential effects on the common ALP-mediated pathway. Importantly, the mixture experiments demonstrated that combinations of multiple pesticide residues could also be translated into distinct collective fluorescence responses, providing direct support for the feasibility of multicomponent residue screening.

The applicability of the platform was further validated in a honeysuckle matrix, where satisfactory recoveries and precision were obtained for TCE-spiked samples. These results demonstrate that the ALP-Cu^+^-click-CRISPR cascade can operate in a chemically complex plant-derived matrix and retain its analytical performance.

Overall, the proposed strategy establishes an effect-based collective sensing paradigm for preliminary screening and early warning of pesticide contamination. It is intended to complement rather than replace compound-resolved analytical techniques. Future studies should focus on improving assay efficiency, expanding interference and matrix validation, establishing systematic mixture-response models, and integrating the platform with chromatographic reference methods to further evaluate its applicability to real-world multiresidue analysis.

## Figures and Tables

**Table 1:** Spike recovery test of TCE in honeysuckle extract.

Spiked (mM)	Recovery 1 (%)	Recovery 2 (%)	Recovery 3 (%)	Mean (%)	RSD (%)
0.2	96.45	103.02	107.06	102.18	5.24
1.0	103.43	98.25	107.57	103.08	4.53
2.0	92.33	94.29	98.40	95.01	3.26

## Data Availability

Data will be made available on request.
